# Gendered norms of responsibility: reflections on accountability politics in maternal health care in Malawi

**DOI:** 10.1186/s12939-018-0848-3

**Published:** 2018-09-24

**Authors:** Elsbet Lodenstein, Kyra Pedersen, Kondwani Botha, Jacqueline E. W. Broerse, Marjolein Dieleman

**Affiliations:** 1 Athena Institute for Research on Innovation and Communication in Health and Life Sciences, VU University and Royal Tropical Institute (KIT), P.O. Box 95001, 1090 HA Amsterdam, the Netherlands; 2 VU University, Amsterdam, the Netherlands; 3 Foundation for Childrens’ Rights (FCR), Mzuzu, Malawi; 4 Athena Institute for Research on Innovation and Communication in Health and Life Sciences, VU University, De Boelelaan 1085, 1081 HV Amsterdam, The Netherlands

**Keywords:** Maternal health, Sexual and reproductive health and rights, Accountability, Gender, Power relations, Traditional authorities, Governance

## Abstract

**Background:**

This paper aims to provide insights into the role of traditional authorities in two maternal health programmes in Northern Malawi. Among strategies to improve maternal health, these authorities issue by-laws: local rules to increase the uptake of antenatal and delivery care. The study uses a framework of gendered institutions to critically assess the by-law content, process and effects and to understand how responsibilities and accountabilities are constructed, negotiated and reversed.

**Methods:**

Findings are based on a qualitative study in five health centre catchment areas in Northern Malawi. Data were collected using meeting observations and document search, 36 semi-structured individual interviews and 19 focus group discussions with female maternal health service users, male community members, health workers, traditional leaders, local officials and health committee members. A gender and power sensitive thematic analysis was performed focusing on the formulation, interpretation and implementation process of the by-laws as well as its effects on women and men.

**Results:**

In the study district, traditional leaders introduced three by-laws that oblige pregnant women to attend antenatal care; bring their husbands along and; and to give birth in a health centre. If women fail to comply with these rules, they risk being fined or denied access to maternal health services. The findings show that responsibilities and accountabilities are negotiated and that by-laws are not uniformly applied. Whereas local officials support the by-laws, lower level health cadres’ and some community members contest them, in particular, the principles of individual responsibility and universality.

**Conclusions:**

The study adds new evidence on the understudied phenomenon of by-laws. From a gender perspective, the by-laws are problematic as they individualise the responsibility for maternal health care and discriminate against women in the definition and application of sanctions. Through the by-laws, supported by national policies and international institutions, women bear the full responsibility for failures in maternal health care, suggesting a form of ‘reversed accountability’ of women towards global maternal health goals. This can negatively impact on women’s reproductive health rights and obstruct ambitions to achieve gender inequality and health equity. Contextualised gender and power analysis in health policymaking and programming as well as in accountability reforms could help to identify these challenges and potential unintended effects.

**Electronic supplementary material:**

The online version of this article (10.1186/s12939-018-0848-3) contains supplementary material, which is available to authorized users.

## Background

In Malawi, like elsewhere in Sub-Saharan Africa, traditional authorities have an important role in rural communities [1]. A study by Afrobarometer [2] finds that traditional leaders are highly regarded and trusted by the public, in particular for their function in dispute resolution and conflict mediation. Scholars argue that traditional leaders, like families and religious institutions, remain the primary locus of political obligation and moral imperative in many communities in Africa [3]. They have unique capacities in brokering relations of individual and collective responsibility and accountability in order to safeguard social norms and promote community mobilisation [3–5]. Furthermore, development organisations have shown an interest in the potential contribution of chiefs to public service delivery. Their involvement in the health sector, for example, is expected to increase local ownership of health services, socio-cultural relevance and sustainability of health programmes [6] and the adoption by communities of public health norms [7]. The position and relevance of chiefs remain highly debated, however, whereby critics charge that chiefs impede inclusive development as they are unelected and therefore undemocratic and unaccountable [8]. Existing studies conclude that much remains unknown on how traditional leaders manage or influence local accountability relations [3, 9]. This paper aims to provide insights into the role of traditional authorities in two maternal health programmes in Northern Malawi. It examines how chiefs mediate and formalise the distribution of responsibilities and accountabilities in communities in efforts to increase the uptake of antenatal and maternal health care. The paper critically assesses the gendered nature of this distribution process and the impact on women’s maternal health and rights. It discusses the implications for the way in which we understand accountability and for the approaches used in policy-making and programming in the area of sexual and reproductive health.

Chiefs in Malawi are empowered through the Constitution and several legal Acts, and they are members of local governments under the 1998 decentralisation policy (see also Additional file 1). They can establish by-laws, referring to rules and norms to regulate life in communities and to levy fines for the non-compliance to those by-laws. For example, some chiefs can oblige community members to contribute their labour to construction work or development activities. If members do not participate, they may be fined [4]. This fine-based system also applies to other sectors, including reproductive health. The use of fines for home-based deliveries has been in place since the 2007 ban on traditional birth attendants (TBAs) whereby chiefs could impose sanctions on women who delivered with a TBA [10]. The successful reduction of the maternal mortality ratio in Malawi, from 957 deaths per 100,000 live births in 1990 to 634 deaths per 100,000 live births in 2015, is often attributed to the community involvement approach and in particular, the fine system used by local chiefs [11]. The application of fines for home births is not unique to Malawi; it is reported in Zambia [12], Burkina Faso [13] and Tanzania [14], among others. In an evaluation of strategies to increase health facility deliveries, Butrick et al. [11] suggest that a fine-based system is generally considered appropriate because it is more affordable for a developing country like Malawi, than an incentive system (e.g. cash transfers), applied in many other countries. Furthermore, it is assumed that, because fining within the chiefs system in Malawi is embedded in traditional ways of regulating social behaviours, fines for women in the field of maternal health are socially acceptable. Health system actors may also justify penalties as a fair response to home delivery as communities have been educated about the importance of facility delivery [12]. Some authors, however, raise critical questions about the use of penalties as incentives to influence health-seeking behaviour. Penalties for home deliveries are regarded as illegal and unethical and as an impediment for women to attend health services. In particular poor women, who tend to deliver at home, may face additional financial hardship because of the penalties, exacerbating health inequities [12, 15]. Authors have warned of potential unintended effects of well-intended programmes to reduce maternal mortality [12, 13, 16]. Despite these contestations, by-laws continue to be used in reproductive health programmes, such as in the Male Championship and Safe Motherhood programmes in Malawi.

The Male Championship Programme is implemented by the Ministry of Health, with support from UNICEF. It was piloted and scaled up in the Northern Region since 2012. In this programme, selected men, called ‘male motivators’, are tasked with encouraging men to accompany their wives to antenatal clinics and HIV Testing and Counselling (HTC) to prevent Mother-to-Child-Transmission of HIV and to encourage their wives to deliver at a health centre. As part of the programme, chiefs were invited to develop strategies to promote compliance with the male involvement initiative [17]. The Presidential Initiative for Maternal Health and Safe Motherhood, initiated by then-president Joyce Banda in 2012, aimed to reduce maternal mortality. The initiative included the establishment of safe motherhood committees, ‘secret mothers’ who discretely monitored pregnant women, the training of community midwives and the construction of maternity homes [11]. The programme emphasised the need to involve traditional and religious leaders. It aimed to engage, train and incentivise the 20,000 village chiefs in Malawi to take the lead in changing attitudes and perceptions regarding maternal health at grassroots level [18, 19]. The first programme proposed chiefs to implement local by-laws that involve punishments for women who fail to bring their husbands to antenatal care (ANC) while the second programme proposed the use of by-laws for delaying ANC as well as delivering at home [11, 20]. In this paper, the by-laws will be further explored using an analytical framework of gendered institutions. With this framework we aim to uncover the underlying local power dynamics in negotiations over responsibilities and accountabilities in maternal health care.

## Methods

### Theoretical framework

According to the definition of accountability by Brinkerhoff and Wetterberg [21], by-laws can be considered an accountability tool whereby a pregnant woman or her partner have the obligation to provide information about, and/or justification for her/his actions in response to the chief who has the power to make those demands and apply sanctions for non-compliance. This form of accountability, however, reverses roles as common understandings of accountability are about the obligation of agents (powerholders), rather than subjects (subordinates or groups of people that are individually less powerful), to take responsibility for their actions [22, 23]. We rather define by-laws as a social process of the translation of norms and rules that involve a “collective expectation of behaviour in terms of what ought to be; a collective expectation as to what behaviour will be; and/or particular reactions to behaviour, including attempts to apply sanctions…*”* (Gibbs, 1965: 589) [24]. Norms express particular values and relations of power, and they are gendered in the following ways:

1. in their *formulation*, they articulate societal expectations regarding the roles, behaviours and attitudes that are considered appropriate for men and women; they may privilege either men’s or women’s interests, such as in the case of male involvement or gender quota in local elections;
2. norms are always subject to *interpretation* and are developed, accepted, maintained, circumvented, manipulated or contested by *actors* who are operating according to hierarchical power relationships, based on gender (among others);
3. in practice (*implementation)*, they are applied differently to men and women, and different groups of women; and
4. they have differential *effects* for men and women (e.g. health outcomes or gender equality outcomes) [25, 26].

By studying the by-laws as a social process of norm formulation and implementation from a gender perspective, we aim to understand how responsibilities and accountabilities are constructed, negotiated and shifted.

### Study setting

Data collection took place between April and June 2015. The study was coordinated by a Dutch researcher and conducted in collaboration with a Malawian non-governmental organisation working on maternal health care and Dutch and Malawian research assistants.

Mzimba district is divided in ten Traditional Authorities (governed by chiefs); out of these, two were selected for the study; they had been part of the project of the partnering organisation. In the first instance, one research site (health facility and its catchment area) was selected where participants were purposefully selected to represent the diversity of views on community participation in maternal healthcare. Because one health facility only has two skilled health workers, we included four more study sites (four health facilities) for additional data collection. Table 1 provides some maternal health data of the study sites involved.

**Table 1:** Basis statistics of health centre study sites

Facility	Births (annually)	Maternal deaths (annually)	Referrals (annually)	Population	Skilled staff		Community health workers		Beds
	2014	2014	2014	2015	MA^c^	NMT^d^	HSA^e^	HA^f^	Labor
District^b^	313	0.22	13	385.482 (2012)					
HF1	593	0	11	45.000^a^	1	2	10	5	2
HF2	220	1	2	6.000^a^	0	1	7	N/A	2
HF3	240^a^	N/A	N/A	7.000^a^	0	1	N/A	N/A	1
HF4	298	0	6	20.000^a^	1	1	5	4	2
HF5	311	0	10	10.441	1	1	5	N/A	N/A

Data provided by the Health Management Information Systems of the District Health Office

^a^Estimation by facility manager

^b^On average, northern part of the district

^c^Medical Assistant (clinician)

^d^Nurse-Midwife Technician

^e^Health Surveillance Assistant (Community health worker)

^f^Hospital Attendant/Assistant

### Data collection

According to the authors of our theoretical framework, norms are expressed in *rules* (informal conventions as well as formal procedures), *practices* (behaviour), *narratives* and *mechanisms of enforcement* (which may consist of an actual sanction but also of arguments why a norm is to be held valid), which can be observed by researchers. Formal rules are conveyed through documents, practices are conveyed through examples of implementation and narratives are transmitted through storytelling and symbols [26]. We collected data through document search, observation and semi-structured interviews as well as focus group discussions (FGD) to convey points of agreement and contestation regarding norms in maternal health care.

Where available, documents such as minutes of meetings (health committee, local government) and texts on the by-laws were collected. In one Traditional Authority a large meeting took place on by-laws during the study period; the main researcher (author 1) used it to observe interactions, collect information on the formulation of by-laws and to talk to local chiefs and local government councillors. Interviews and FGDs took place with participants of different groups involved in maternal health care.

The interview and FGD guidelines included four main topics: (1) experiences/engagement with maternal health services; (2) perceptions on the quality of care; (3) community participation and responsibilities in maternal health care; and (4) priorities for better maternal health care. In the FGDs, participants were given additional exercises to discuss challenges in the organisation and quality of maternal health care and to discuss responsibilities and accountabilities. The researchers encouraged discussions through probing questions *(*e.g. *“who do you think is responsible for improving maternal health in this community?”; “if you were to introduce a code of conduct for health workers and service users in this health centre, what would be the most important duty you would formulate?”).* The term “by-law” was not explicitly part of the interview guidelines but emerged from the participants as they shared their perceptions on responsibilities in maternal health care under topics 3 and 4 of the interview guideline and in the FGD.

Interview and FGD guides were translated from English to Tumbuka, back translated and tested with key informants familiar with interviewing techniques in the research area. Interviews lasted between 50 and 60 min, and FGDs ranged in size from 5 to 8 participants and lasted on average 1.5 h. Only the interviews and FGDs with health workers were held in English; a translator assisted the others. One researcher (author 2) conducted most individual interviews whereas two researchers (authors 1 and 2) conducted the FGDs together or separately.

### Study participants

Table 2 presents the type and number of study participants included in this study. The final number of participants is 137 of which 36 participated in individual interviews and 101 in FGDs. Participants included 35 female maternal health service users of which three were guardians (women escorting pregnant women to the health centre for childbirth); 19 men with experience with the health centre as escorts or as husbands; 25 health workers, including auxiliary staff, health surveillance assistants and facility managers; 34 community leaders (of which 24 local government representatives and 10 chiefs); 20 community representatives of the health committee and 4 key informants (from the district health office and non-governmental organisations). Female representation in the health worker group was 36%, in the community leaders group 25% and in the health committee group 50%.

**Table 2:** Number and types of participants per study site

Method	Participant type		# participants TA 1	# participants TA 2	District level
# of health centres covered			3	2	
In-depth individual interview	Women (service users) and guardians		8		
	Health workers		6		
	Health committee members		4		
	Chiefs		4	6	
	Local government		2	2	
	District health office				2
	Key informant (NGO)				2
Focus group discussions	Women (service users)	5 FGD	22	5	
	Husbands	4 FGD	14	5	
	Local government	2 FGD	8	6	
	Village development committee	1 FGD	6		
	Health committee members	3 FGD	4	12	
	Health workers	4 FGD	12	7	
Total # participants			90	43	4
Meetings	By-law meeting +/− 60 participants (70% male, 30% female)				

In both Traditional Authorities, local government secretaries helped the organisation of the interviews and FGDs with members from local government, health committees, village development committees and chiefs. The researchers contacted health facility managers directly and informed them about the research. Women were identified with the assistance of health surveillance assistants or at the health centre when attending postnatal services; they were asked to participate in an interview or FGD. A point of saturation approach was applied to the sample of all participants when the range and distribution of actors, views and experiences were covered in different sites and when the researchers expected no further insights to emerge. The sample of chiefs is small; in particular the views of different levels of chiefs may be underrepresented. For example, it was hard to recruit the higher level of chiefs for individual interviews. Six chiefs were briefly interviewed during the by-law meeting; the interviews were not recorded and did not follow the interview guidelines of the other groups but focused on the by-laws.

### Data analysis

Interviews, FGDs, and the by-law meeting were recorded, transcribed and translated into English by research assistants and checked by the researchers. Transcripts and collected documents were introduced and analysed in Maxqda (Version 11). The first step of the analysis involved exploring the (type of) information provided in each data source and using a few transcripts from different participants to explore stories, their contexts and variations as well as the terms participants used to talk about responsibilities and accountabilities in maternal health. In the second step, we extracted segments relating to the by-laws under one general code and later categorised them according to the topic of the by-laws (ANC, male involvement and institutional deliveries). A third step involved the identification of themes (main codes) using the four dimensions of the theoretical framework: formulation, interpretation, implementation and effects, that contained segments on the background, descriptions, perceptions and accounts on the implementation of the by-laws by participant group. We used the notes and report of the by-law meeting to identify the wording of the by-laws and the main points of discussion. Results of this third step of analysis are presented in the results section. The fourth step involved the analysis of the gendered nature of the by-law process and effects. For this, we applied a gender analysis to the coded segments. This was followed by a separate analysis (step 5) of the actors involved across the themes of formulation, interpretation and implementation. Data were first analysed and collated per participant group and then compared to identify gender differences in answers. A description of data coding and analysis questions for steps three to five is provided in Table 3. The results of the gender and actor analysis are mainly reflected in the discussion section.

**Table 3:** Coding framework and steps of analysis

Main code	Description (step 3)	Gender analysis (step 4)	Actor analysis (step 5)
1. Formulation of by-laws	Identifiable *rules* - official, legal or widely known or reported (written and unwritten) regarding expected and sanctioned behaviour.	Rules about gender within the by-laws (how are the responsibilities of women and men formulated; what are the defined sanctions for women and men?).	Who participates, who develops the content of the by-laws, who enforces?
2. Interpretation of by-laws	*Perceptions* about the objectives and appropriateness of the by-laws.	Perceptions about gender roles and responsibilities in maternal health care; perceptions about the by-laws as a tool to correct behaviour in maternal health care.	Who confirms, accepts, questions the rules; how are actors positioned in the gender hierarchy? The interaction in FGDs was analysed separately (what topics produced consensus or conflict; whose interests were being represented; was a particular member silenced?).
3. Implementation and effect of by-laws	Evidence of implementation (issuance of sanctions) and its effects on the actors involved.	The gendered implementation and effects of the by-laws.	Analysing specific accounts: to whom and under which circumstances is the by-law applied; what were decisions involved and who made the decisions; who is affected by the application of the by-law and how?

## Results

The results are organised according to the themes of the analytical framework: formulation (pertaining to the content of the by-laws), interpretation (pertaining to participants’ perceptions of the by-law), implementation and effects (pertaining to actual instances of application) of the by-laws.

### Formulation of the by-laws

In all study sites, chiefs play a role in the development and regulation of social and economic activities. They often mobilise communities to mould bricks and build clinics and health staff housing or shelters. They also address crime, conflicts, and illegal activities such as unauthorised logging. Many chiefs have introduced by-laws establishing, for example, the obligation of families to build latrine pits or to send their children to school. According to study participants, not all chiefs actively monitor the rules and apply the sanctions as their authority and legitimacy vary. Recently, decentralised local governments have become interested in strengthening the coherence between by-laws from different areas and in scaling up their implementation to support national policies. The local governments in the study area had consulted chiefs at the community level and village development committees to identify the main development problems for which by-laws could be established. Each of those community consultations mobilised around 100 participants of which 40% were women. A by-law meeting at the level of the Traditional Authority was meant to aggregate data and prioritise the by-laws, refine the rules, sanctions and enforcement mechanisms. Criteria for prioritisation were the level of complementarity with other laws and measures taken by the government.

Three by-laws on maternal health were adopted at the meeting: the first requires mandatory ANC visits by women from their third month of pregnancy; the second obliges men to escort their wives to the ANC clinic; and the third obliges women to give birth in a health centre. This reflected priorities from the community consultation meetings, although other sexual and reproductive health issues had come up there, such as child marriage, teenage pregnancy, confidentiality of HIV testing and sexual harassment in schools. Compared to other by-laws, for example on hygiene and sanitation or child education, those concerning maternal health were only briefly discussed as they were considered clear for everyone, institutionalised, grounded in national policy and applied in most villages. The consensus at the meeting was that these by-laws did not need a redefinition or re-enforcement.

All groups of interview and FGD participants mentioned the existence of by-laws on maternal health. However, they formulated the content of the by-laws differently from each other and from the definitions in the meeting. The most common formulations are presented in Table 4. Non-institutional childbirth, including home-based deliveries, childbirth on the way to the hospital and late arrival at the health centre were all punishable acts. Punishments ranged from capital (animal) to financial penalties. The by-law on male involvement in ANC services provides a condition under which unescorted visits are allowed: women need to justify the absence of their husbands by presenting a letter from the chief to the health facility.

**Table 4:** Rules regarding ANC and institutional childbirth

Antenatal care visits *“When a woman is three months pregnant she should start going for ANC; if she fails she should be fined”* (by-law meeting).	
Male involvement in ANC *“When a man does not escort the wife to ANC there should be a punishment”* (by-law meeting). • This can be avoided if the woman has a letter from the chief justifying the absence of the husband. • A trophy is given out quarterly to a health centre that has performed well in terms of male engagement (District health office/UNICEF)	
Institutional childbirth *“Women will be punished for not delivering in the health centre”* (by-law meeting). *Specific by-laws:* • *“A woman who has gone to a TBA should be punished”.* • *“When a woman delivers at home or on the way there should be a punishment”.* • *“When a woman arrives late at the HC, she should be punished”.* Penalties for home deliveries involve paying a goat or an amount between 3000 (USD 5) and 20,000 MKW (USD 35). Deliveries on the way to the facility are fined with 1500 MKW (USD 2.5) and for late arrivals 1000 MKW (USD 1,8)^a^.	

^a^At the time of the research (2015) the exchange rate was approximately: 565 MWK = 1 USD. The GDP per capita was USD 1200. The household cost (medical and transport etc.) for women admitted to complication care in Malawi was approximately 7 USD in selected districts in 2015 [27]

Data from the interviews and FGDs reveal that chiefs at village and group village level initiate, monitor and enforce the by-laws and they issue and collect the fines. This was confirmed by data from the by-law meeting. Chiefs are informed about non-conforming cases through a network of community-based structures such as the ‘secret mothers’, health surveillance assistants, village development committee members, health committees and health workers. The content and procedures of the rules regarding maternal health were hardly questioned in the by-law meeting. By contrast, reports from the community consultation meetings that were held prior to the by-law meeting and data from the FGDs showed that there were objections to the idea of by-laws, restricting free choice and other human rights. Hence, it seemed harder for people to debate the content of the rule higher up in the hierarchy of local government.

### Interpretation of the by-laws

#### Perceptions of norms addressed in the by-law

The majority of participants fully supported the principles addressed in the by-laws. Participants agreed on the importance of male involvement in maternal health care, albeit for different reasons. From the women’s point of view, husbands, when attending ANC clinics, become aware of the financial and material resources that are required during pregnancy and childbirth. Husbands, women argue, would take the advice on pregnancy and childcare from a nurse and clinician more seriously than from their wives. Husbands confirmed in an FGD that it was important to attend ANC *“to hear what is needed at the hospital”.* Most of the husbands in FGDs had attended training and education sessions by male champions and they were aware of their obligation. Most had accompanied their wives during the most recent pregnancy for financial, material and medical reasons, but a few also saw it as an ‘act of love’. Two others mentioned the reciprocity in their marriage: *“a man takes a wife as a helper and then needs to help back”* and *“she helps around the house and takes care when you are away, so you need to take good care”.* A local government chairman confirmed that the by-laws had led to changed gender relations *“as per culture, we feel like mothers are the ones who should come to the maternity, not the fathers. That has now changed, many men see reproductive health as a joint responsibility”.*

Health workers, in particular, saw the importance of male involvement for birth preparedness and maternal and child survival. They felt the by-laws emphasised that couples should jointly bear the consequences of pregnancy, and in particular, the complicated ones. Health workers and women also shared a strategic interest: men who accompany women during ANC and childbirth *“cannot continue having children when they see the woman’s problem”.*

The need to give birth with a skilled birth attendant was widely recognised among all participant groups to ensure a safe delivery and maternal and child survival. A local leader associated institutional deliveries with the maintenance of families and the future of the community:

*“When expectant women patronise the hospital, come in time…, that means we are sure we’ll have a future as the future lies in the new generation. And if the new generation comes in and is protected, we are sure that it is maintained and developed” (male local government chairman - ADC01–1).*

#### Perceptions on the by-law as instrument to enforce norms

In interviews and FGDs, participants, in particular local leaders and health workers, repeatedly referred to the by-laws as a strategy that had worked best to change behaviours of women and men. The value of having by-laws, according to the participants, lay in their potential to raise awareness on correct and incorrect behaviour, and lead to positive behaviour change. According to a health worker, by-laws on institutional deliveries are effective in that they provide a warning and a sense of fear among women:

*“If that one would be punished then the others can learn a lesson saying eh we are going to be punished like our friend so others will be afraid. So at least they can change their behaviour”* (FGD health workers HF2).

According to the following statement of a health committee member, by-laws draw on authority (the right to punish), political legitimacy (as part of government policy) and democratic legitimacy (instrument of a ‘parliament’) needed to change attitudes and behaviour towards reduced maternal mortality:

*“The chiefs should take part because if it comes from them it carries more weight. Since there are by-laws, a woman should not give birth at home and should not skip her antenatal care days to avoid being fined. Even if it was a mistake to have birthed at home, still the fine will have to be paid. These laws acts as our parliament, and once said it is never reversed. Since the chiefs have taken side on this issue, it has given rise to the number of women coming to the hospital. Even a husband now is part of this issue because they escort their wives to the hospital”* (male health committee chairperson HF1).

Local leaders and health workers considered by-laws on male involvement in sexual and reproductive health crucial, as men are a particularly difficult group to reach through other methods. By-laws were seen as the last resort to get men to comply with instructions from the health educators or male champions and to respect their duties as spouses and fathers. According to a local government chairman, with by-laws, the *“people buy what the government is saying”.*

#### By-laws for intentional non-compliance

The need to have by-laws aimed at women’s health-seeking behaviour was justified to promote compliance with health educators’ instructions on safe motherhood. In the eyes of local leaders and some health workers, punishments were considered particularly important for those women, who, despite health education and information, increased knowledge on safe childbirth and increased access, lacked the *will* to comply to rules. A nurse explains that only intentional actions should be punished:

*“If she just started the labour pains right at the moment when she has come…then there is no need to punish her. To me, I feel like, a punishment maybe could be there for those who have decided “I will never deliver at the hospital, I’ll deliver at home”, Yeah, there, the punishment is right”* (male nurse-midwife – HW05HF4).

One health worker adds that it is the woman’s deliberate choice to delay *“because they start to do other things”*. This point of view is also reflected in the unwavering opinions of a chief and a district health officer. They argue that the rule should universally be applied to every pregnant woman as:

*“Every pregnant woman knows she is about to deliver and she is supposed to go to the hospital before the ninth month”* (Chief – GVH02HF3).

*“They know the policy, and it is due to their own delays that they deliver at home”* (District health officer-DHO01).

The district health officer and local government secretary add that women can avoid the penalties by making use of the maternity waiting homes provided by the authorities. Hence, according to them, women are to be blamed for not using health facilities and for the consequence, the penalty. According to such perceptions, women need to be held to account for their reluctance to attend ANC and institutional delivery services.

An additional argument for punishing women came from a female nurse-midwife who suggests that women should be punished for taking medical risks, but also for putting health workers at risk of losing their license:

*“We should tell her that you did not do good because if something happens like any complication, the ones to suffer will be the health workers, not the woman. If you are the nurse they [referral hospital] will see that complication, and they cannot say that patient came in late, they will just take away your license, and you may be fired. So you agree with the community on ways of punishing that woman”* (FGD health workers HF2).

The proponents of the by-laws and punishments were mainly authorities such as the district health authority and local government representatives, all men, and the nurse-midwife quoted above. Other participants, however, held other opinions on the fairness of the by-laws as discussed in the next section.

#### Contesting views on the fairness of the by-laws

During interviews and in particular during FGDs, some health workers, local government councillors and health committee members (women and men) held nuanced views on the by-laws. They were in particular critical about the prevailing principles of individual responsibility and universality, as illustrated by two case studies (see Table 5).

**Table 5:** Case studies

Case 1: Home births and delays in arriving at health centres: individual versus collective responsibility. There was disagreement among participants on whether it was appropriate to penalise individual women for home births and late arrivals at the health centre or whether families, chiefs and communities should be held accountable. According to two male councillors, the community, in particular the chiefs should be fined as *“it is their job to save mothers”* and they are the ones responsible for the promotion of institution-based deliveries. The anticipation of punishment for chiefs would allow them to increase their efforts and it would be a way to hold them accountable. Others, however, proposed the punishment should apply to the family of the woman, including the husband, as barriers to reach the health facility in time are most likely related to familial circumstances. Moreover, husbands are the ones who need to assist with potential blood transfusion and transport in case of complications and they are directly responsible for maternal survival. A clinician and a nurse did not want to blame the husbands as they usually are willing to release money and join their wives but it is guardians (a pregnant woman’s family member or elderly woman accompanying them to the clinic) who intentionally delay women, and who promote home births. Case 2. Delay in arriving at the health centre: universality versus unique circumstances. Health workers held nuanced views regarding the conditions under which women should be punished or not for arriving late for childbirth. For example, a male clinician and a female nurse argued that exceptions should be made for women living far away from the health centre. Other health workers were of the opinion that punishment should not be routine practice because *“birth cannot be predicted”* and *“there can be miscalculations in due date”.* Another female nurse argued that poor families compare the costs of coming early to the maternity waiting home and coming late and decide to come just before the delivery to save costs. She insists the task of a health worker is to understand the reasons for late arrivals of women before making a judgement. In two FGDs, health workers agreed that they should help women regardless of their timing to attend the facility. Hospital attendants and health surveillance assistants, but also some male and female nurses, emphasise that advising and counselling is better than punishing since this fits better the potential personal circumstances of women as well as facility circumstances, as explained by a male health worker: *“The way it is here, the maternity ward is small, and many people also sleep on the floor, it’s your luck sometimes that a person comes when it’s already time to give birth. She has left the space for other women, others come from faraway places, so you see she lightens your burden in that she will give birth just three days and she is out”* (male environmental officer HF1).	

During the FGDs with health workers, the researchers observed the tendency of health surveillance assistants and support staff to be more forgivable towards women and hence more critical towards the by-laws. It was only during the discussions that nurses and clinicians joined this position leading to a common agreement that by-laws did not fit the reality and complexity of maternal health care.

### Implementation and effects of the by-laws

According to participants’ accounts, the by-laws are unevenly implemented. Some chiefs do not monitor and enforce them, some do it very strictly; some health workers continue helping women despite coming alone to ANC or late for their delivery, and some health workers are applying self-made sanctions. Table 6 presents material and non-material sanctions that were applied for non-compliance to the rules of maternal health care. Non-material sanctions include women’s experiences and feelings of being discriminated and unfairly treated by leaders and health workers.

**Table 6:** Implementation of the by-laws

Rules	Material and non-material sanctions reported
Antenatal care visits	• Denial of care by health workers • Financial penalty issued by health worker for coming late to ANC (beyond three months)
Male involvement in ANC	• Chief refusing to write letter to excuse absence of husband • Women coming without husbands treated after women who are with husbands • Women who come alone sent back by health workers • MoH and UNICEF introduced a new award that health facilities can win if they increase the number of male ANC attendants
Institutional childbirth	• Payment in kind for home delivery (goat) • Financial penalty for home delivery ranging from MKW 6000 to 20,000 (USD 10–35) • Verbal insults to women who do not bring material, such as razors, soap and cloth, to the facility for their delivery

Interestingly, all sanctions applied to women were negative sanctions, while one positive sanction – an award – was introduced to reward health workers for their efforts to increase male involvement, illustrating how behaviour of women and health workers is valued differently.

#### ANC visits

Rather than chiefs monitoring and applying the by-law on ANC visits, health workers are reported to punish women who do not attend ANC or do so at a later stage in the pregnancy. A female ADC member attested:

*“As they are three months pregnant, they want to start the ANC but sometimes they are delayed. When they go in the fourth month to the hospital, they are not assisted and they are fined to pay maybe 2,500 (USD 4). So they go back home to try to find some money, and even if they do not find money, they still go the health facility to not be further delayed. They [health workers] will end up shouting at you in bold language, “how come you didn’t find the money!”. And even those who pay the money are delayed and it does not become good”* (female local government councillor in FGD – W3FGDADC1).

#### Male involvement in ANC

The by-law that requires women to justify the absence of their husband in writing is actively applied in some villages but not in others. Local government councillors reported that the by-laws are often not followed; that some chiefs do not have the power to admonish husbands or the capacity or will to write a letter. They also indicated that the safe motherhood committees do not follow-up on the by-law and sometimes even discourage men to accompany their wives, as some members consider male involvement in ANC as socially and culturally unacceptable. A chief, however, states he applies the rule and has husbands pay a goat for their absence during ANC. Where a chief is not active, health committee members sometimes follow women home to ask the husband why he did not come as they feel co-responsible for maternal health.

Local government chairmen from both Traditional Authorities report they receive many complaints from the community about the requirement to have a letter to justify the absence of husbands. Such complaints come from women whose husbands work abroad or whose husbands refuse to engage in maternal health care. A local government councillor described two cases whereby the chief refused to write a letter because the husband refused to escort his wife. An elderly woman even appealed to excuse the woman for having a defiant spouse and to grant her the letter. A health worker reported that it was particularly difficult for unmarried women, and for women with an absent or refusing husband to get a letter from the chief. These women, consequently, were more likely to give birth at home. A local government councillor narrated one such case:

*“A baby died here last year. The woman told the husband to go to antenatal care, but he refused. Then she came alone, but without the letter, the health workers sent her back. The second time she came alone again, and they sent her back again. At last, she just sat down until she delivered at home, her mother tried to get her to the hospital, but she delivered right at home. The baby was also born with diseases, the baby stayed for two days and died because the husband was negligent”* (female local government councillor in FGD - W3FGDADC1).

It is striking in this case that the blame for a neonatal death is placed on the husband for being ‘negligent’ rather than the health facility staff for refusing to provide care. Another councillor confirmed receiving complaints of similar cases whereby women are being sent back when they come alone for ANC. The denial of services by health workers then constitutes an additional punishment not foreseen in the by-laws. Understandably, this also works the other way around; there are also cases where women are helped in health centres, despite the fact that they are alone. Health workers then consciously ignore the by-law, or do not actively take advantage of it for their own interest.

#### Institutional childbirth

It was unclear from the interviews to what extent the by-law on institutional childbirth is enforced. One participant said that since it has existed for some years, the by-law works now as a reminder; institutional deliveries are *“in everybody’s mind and hearts”.* Most women in FGDs stated they had not experienced paying a fine themselves but that they had heard it was applied. One health worker also stated he has never seen a woman paying a goat, while six health workers in an FGD all had seen women paying different amounts, mostly around 6000 MKW (USD 10) equalling the price of a goat. Reports from health committee members, confirmed this. Two members explain that they report *“women who have broken the law”* to the chiefs and that goats are being paid. Apparently, variations exist whereby health workers administer penalties as they feel is appropriate to women who do not comply with maternal health instructions. A male nurse, for example, explained that they ask women who come late for their delivery (when labour already started) to pay some money to women in the maternity waiting home as to relieve them as a form of solidarity fund. A health committee member reports another form of punishment for not complying with the rule to bring material for the delivery:

*“Those who come empty-handed, or without the disposable black paper or the razor blade, the doctor finds the items for them. But once they have delivered, they are given a punishment…they will not be discharged unless they pay back the items”* (two male health committee members HF2).

Women also saw verbal abuse and health workers’ shouting at them as a form of punishment for not bringing material. Women justified this punishment as they blamed themselves for not obeying the rules.

## Discussion

This study provides a detailed account of the content, process and effects of by-laws in the field of maternal health care in Northern Malawi. A first main finding is that in the study area, the by-laws on maternal health are widely known, discussed, applied and even scaled up by local governments. They reflect by-laws in use in other districts in Malawi [16, 17]. The findings of this study show how they are a visible feature and reality of everyday lives of women and men in the community and a discernible element of the health system. The second main finding is that, through the system of by-laws, chiefs and local authorities have considerable influence over who accesses services and on what terms. Hence, they are important actors in the complex web of accountability relationships in the local health system. These actors, however, operate in a gendered environment that is reflected in the ways in which actors construct, negotiate, legitimise and contest women’s and men’s behaviour, responsibilities, accountabilities and criteria for sanctions. This paper does not aim to debate the position of traditional leaders in society but its value lies in providing a critical assessment of the gendered nature of local governance processes, with by-laws as central features of maternal health care in Malawi. In the following, we will elaborate on the gendered nature of the by-law process as well as the implications for the way in which we understand accountability and for the approaches used in health policy-making and programming.

### The gendered nature and process of the by-laws

While we organised the results section in three separate steps of the by-law process, we recognise that the steps interact and reinforce each other when it concerns gender and power issues.

In their *formulation*, the by-laws are gendered in that their subjects are individual women who are (threatened) with punishment and in many cases punished for not complying with the instructions for safe motherhood defined in policies, and social rules defined in communities. The gendered relations of responsibility and accountability are particularly visible in the by-law on male involvement. While the *responsibility* of husbands in maternal health care is officially defined and increasingly accepted socially, the *accountability* is claimed from women through a range of sanctions for the absence of their husbands. The husbands, and other male community members, are, apparently, more easily excused from their responsibilities. The attribution of individual responsibility to women for successful care and good maternal health outcomes, confirms the fact that reproductive health, and maternal health, in particular, has historically been seen as a woman’s issue or responsibility rather than a right [28]. Also, the attribution of responsibility to women reflects gender as a relation of power, whereby women’s roles are defined in relation to, and after, men’s roles.

The gender bias in the by-laws is problematic for several reasons. The ‘excuse letter’ makes women unwillingly dependent on men, and in particular male partners and chiefs. It disproportionally affects unmarried women or women who have an absent or refusing partner. These women, who may already suffer from discrimination, experience an additional burden in terms of time needed to look for support and funds, the need to plead for a letter from the chief, the potential humiliation in the community and the health centre and delayed services with all the risks involved. A study in Uganda shows how women without a partner even adopt strategies to circumvent punitive measures by taking men other than their partners to the clinic [16]. The discrimination of women on multiple grounds (by gender, marital status, income) in male involvement programmes is confirmed in other studies on Malawi [29] and highlights that gender intersects with other social stratifiers that shape experiences of vulnerability, accountability relations and health outcomes [30].

Furthermore, the attribution of responsibility for male involvement to women ignores addressing particular challenges men face in access to sexual and reproductive health services, such as negative provider attitudes towards men and male unfriendly services [31]. Also, by separating out women from other actors in the by-laws and health programmes more generally, structural barriers of access to care, such as financial and geographical barriers and supply-side factors (availability and quality of human resources and equipment) at the point of service delivery, are overlooked. In sum, in addition to the disempowerment of already marginalised women, the by-laws may result in missed opportunities to achieve programme goals, such as uptake of family planning, HIV testing and PMTCT services and opportunities to strengthen gender equality in families, communities and health systems.

With regard to the *interpretation* of the by-laws, we observed significant differences in perceptions on the appropriateness and fairness of the by-laws including concerns about gender equality and women’s human rights. This was particularly illustrated by the two case studies. Broadly speaking, the actors in our study can be positioned on a spectrum of agreement with the by-laws. On one end, those in authority (such as the local government councillors, secretary, chiefs and some health authorities) legitimise the coercive character of the by-laws through a language of blame (for women’s reluctance to use services) and punishment of (disobedient) women. They emphasise women’s reproductive duty in the family and the community and the duty of community leaders to oversee this role. Some members of local government as well as women and men in communities and those at the frontline of service provision, in particular lower cadres of health workers, are positioned on the other end; they avoid these terms, and consider and defend women’s particular circumstances. The position of health workers vis-à-vis the by-laws seem influenced by their position in the hierarchy whereby those higher in the hierarchy were more rigid and punitive, while those lower down were more lenient and compassionate emphasising their moral obligation to deliver care. We did not observe major differences in opinions by gender or position of technical staff (clinicians or nurse-midwives).

The diversity of views and nuanced opinions contradict the narrow and gender biased formulation of responsibilities and accountabilities in the formal by-laws, which were set during the by-law meeting. During the by-law meeting, the position of persons in authority dominated the formulation and prioritisation process where there was limited space for the nuanced opinions and contesting voices. Moreover, even when concerns about dignity and human rights would have been heard, they might have been neglected in the name of gains in effectiveness of the safe motherhood and male involvement programmes [32]. This shows that international development organisations, through their priorities and preferences, can significantly influence or override local implementation processes, choices and accountability systems [30].

Regarding the *implementation* of the by-laws, we observed important variations in practice and the non-application or selective application of the by-laws. This finding was also reported by Greeson et al. [12] in the context of penalties on institutional deliveries in Zambia. This may partly be explained by the limited knowledge, capacities and authority of chiefs and local authorities to use and enforce by-laws but also by the different interpretations of the by-law. Health workers’ ambivalence vis-à-vis the by-laws seems to translate to some health workers actively supporting their enforcement, some applying their self-defined sanctions and others refraining from applying them or ignoring them. This suggests that health workers have significant gatekeeper power regarding access to services, regardless of local or national regulations and their interpretations [33]. The findings show that such power can turn out positively or negatively. The denial of care by providers or verbal abuse as retribution for not obeying facilities’ instructions has been observed in Malawi before [34]. It illustrates the power asymmetry between women and health workers and fits the larger culture of disrespect and abuse in maternal health care [35]. The gendered accountability relations expressed in the by-laws may strengthen this power imbalance at the frontline of service delivery, disempower women and impinge on women’s right to health, dignity, non-discrimination and non-coercion, among others. Apart from a violation of Constitutional rights, these practices contradict Malawi’s commitments expressed in the Safe and Respectful Childbirth Charter, the Sexual and Reproductive Health Rights policy and the 2013 Gender Equality act, among others [36, 37].

In the following, we will discuss the implications for the understanding of accountability relations and processes, for working with traditional authorities and for the design of reproductive health programmes.

### Implications

#### Understanding accountability relations as gendered

This paper challenges common understandings of accountability in public health literature. Accountability usually refers to the accountability of public actors, ‘the state’ or service providers, while the local accountability dynamics in our case study indicate a form of ‘reversed accountability’, of women towards national and global targets. Similarly, women seem to be held accountable for health workers’ performance and careers instead of the other way around. This important finding shows how gender norms (and other factors dividing power) may disproportionally place the burden on women in governance arrangements and maintain or reinforce their role as passive citizens in whose name programmes are designed and power exercised [38]. Such a role, obviously, reduces women’s ability to hold state and non-state actors (including traditional authorities) to account. Our findings add to the complexity of the task of defining and distributing responsibilities and accountabilities in health systems and reiterate the need to critically assess power asymmetry in accountability mechanisms, including the possibility of such asymmetry to develop into unwanted directions such as the ‘demotion’ of citizens or service users from agents to subjects of accountability.

On a positive side, the case studies in this paper suggest that responsibilities and accountabilities are constantly negotiated, and that this provides space for discussing, questioning and changing gender norms and developing gender-responsive accountability mechanisms. The FGDs in this research project provided a space to discuss the central questions in accountability: who should be responsible for what and under which circumstances?; who has the right to call someone to account and are responsibilities and the accountability process agreed beforehand by all parties?; is a control-based accountability mechanism the right approach to influence human behaviour and what are the alternatives? Social accountability projects in Malawi, and elsewhere, that promote maternal health by building relationships of co-responsibility, could also offer such space [39]. Gender sensitive spaces, then, should go beyond promoting women’s participation but also aim at more substantive change by taking into account power asymmetries and by including gender equality as an object of accountability of all actors, including traditional authorities [40]. It is then that accountability can contribute to equity in access and quality of care and to the realisation of health rights.

#### Working with traditional authorities

As we have seen in this study, the use of traditional forms of accountability based on the assumption that they represent a moral and representative institution risks confirming and reinforcing gender norms and power relations. By leaving the implementation of programmes to community leaders, some targets may have been met but we observed the potential negative impact on (marginalised) women and health equity. We argue that community leaders act in a gendered environment and relying on them to achieve gender equality may be ineffective. But rather than dismissing traditional leaders for their potential disruptive role in service delivery, we argue that more attention should be paid to the way power is exercised and monitored. If substantial functions are transferred from the state to traditional authorities (or other non-state actors or local governments), adequate accountability mechanisms must be guaranteed to justify decisions and actions and avoid abuse of power [41]. This supports Kelsalls’ findings that, although chiefs are criticised in African communities, people want more accountable chiefs rather than no chiefs at all [3].

#### Gender analysis in health research, policy and programming

The literature on health reforms has focused on issues of performance to the neglect of process [32], and evaluation studies in the field of sexual and reproductive health tend to report on the positive intended effects of programmes. Indeed, the Malawian male involvement programmes’ first objective was to improve SRH indicators and evaluations report on positive results regarding access to family planning, uptake of HIV testing and PMTCT services [16, 20]. Participants in our study also reported positive effects of the use of by-laws, such as increased awareness on maternal health issues and gender roles in families and increased uptake of services by women and men. Studies elsewhere in Malawi show that by-laws initiated by traditional leaders have also positively contributed to improvements in other fields, such as child marriage [42]. The current study highlights that it matters not just whether strategies are achieving what they were intended to accomplish (e.g. increased facility-based deliveries), but also how they are enacted and whom they benefit and exclude on paper and in practice. Negative effects, such as the ones described in this study, are likely to have passed unnoticed or when they are noticed, are labelled as ‘unintended negative effects’. Most development agencies acknowledge the occurrence of unintended outcomes, but guidelines on how to detect and evaluate them remain limited [43]. We argue that the gender dimensions of sexual and reproductive health programmes can be detected, evaluated and responded to in programmes through gender analysis. This would help to understand the power dynamics and potential barriers women and men face in accessing services, and to reflect on how a programme can achieve service delivery as well as gender equality goals or at least ensure that inequality is not perpetuated [44]. At the programme level, such analysis would help to compare approaches to motivate women and men to participate in health services and identify approaches that meet (different groups of) women’s needs and respect their rights. The analysis in this study, for example, suggests that the empowerment of community health workers or lower level cadres of health workers may be more effective for women than empowering community leaders to influence health seeking behaviour. Importantly, the gender dimensions of governance arrangements and of access to maternal health services vary per local context and per type of service (e.g. ANC, HIV testing, institutional deliveries etc.). Gender analysis helps to identify context specific opportunities for change. From a policy perspective, gender analysis can transform the discourse of rights and strengthen the intrinsic value of human-rights based approaches in programming, as stipulated by national governments, UNICEF and other development organisations [45].

### Strengths and limitations

The variety of views and power relations among actors underscores the importance of in-depth qualitative research methods. The FGD, in particular, provides participants with a platform to share experiences and a way to distil agreement, disagreement, and power dynamics between actors in the local health system. Unfortunately, it was not possible to organise an FGD with traditional leaders, which might have led to additional insights on the actual implementation of the by-laws, among others. The theory-based character of the study may make findings relevant to other maternal healthcare settings, including beyond Malawi. Before the research, the Dutch researchers were unfamiliar with the phenomenon of by-laws on maternal health; although they regularly discussed findings and interpretations with Malawian key informants, they might have overlooked nuances.

## Conclusions

This paper aims to provide insights into local accountability relations involving traditional authorities in the context of two maternal health programmes in Northern Malawi. The findings and discussion contribute knowledge to the field of accountability in the health sector in four ways. First, the current study complements the literature on the phenomenon of by-laws in maternal health care that is sparsely documented, in particular where it concerns the challenges from a gender perspective. By-laws reflect a power relation rather than an accountability relation and scaling up their use as accountability tools seems inappropriate. Second, it reveals how gendered norms regarding roles and responsibilities in maternal health care play out in practice and impact on health equity. Such norms potentially apply to different types of accountability relations but they are often overlooked in accountability literature. Third, the study confirms the complexity of accountability relations in the context of sexual and reproductive health care and, in particular, the influence of gender inequality in families, communities, maternal health care and health systems on the outcomes of programmes and vice versa. It thereby questions the rationale for embedding health programmes in communities for reasons of sustainability and local ownership. Finally, the paper draws attention to the linkages between global development goals, national donor-funded programmes and local implementing institutions. The emphasis on facility-based childbirth in global and national policies can overshadow other important elements of rights to accessible, acceptable, quality and equitable care, in particular when this emphasis is translated into coercive methods that further impinge on women’s rights. Mainly concerned with states as central targets of accountability, research should incorporate local accountability dynamics and promote a discussion on the emphasis on accountability as a concept and strategy in health programming. Our results also suggest that particular attention should be paid to gender and other power relations in sexual and reproductive health policies and programmes and to potential adverse effects of using local accountability systems.

## Additional file

Additional file 1: Local government structures in Malawi. (DOCX 17 kb)

## Abbreviations

ANC: Antenatal Care
DHO: District Health Office
FGD: Focus Group Discussion
HSA: Health Surveillance Assistant
NGO: Non-Governmental Organisation
TA: Traditional Authority
TBA: Traditional Birth Attendant
