# Supplementary material for: Gendered norms of responsibility: reflections on accountability politics in maternal health care in Malawi
Source: Int J Equity Health. 2018 Sep 24;17:131. doi: 10.1186/s12939-018-0848-3 (PMC6151921; doi:10.1186/s12939-018-0848-3)
Supplement: Supplementary file 1 — Local government structures in Malawi. (DOCX 17 kb) [file 12939_2018_848_MOESM1_ESM.docx]

**Additional file 1. Local governance structures in Malawi**

Due to the adoption of the Decentralization Policy in 1998 and the subsequent enactment of the Local Government Act, most powers of central government were devolved to district administration where chiefs had been drafted into the composition of District Assemblies as *ex-officio* and non-voting members [2]. District Assemblies are responsible for budget and planning decisions, in response to inputs from sub-district structures: Village Development Committees (VCD) and Area Development Committees (ADC), referred to as ‘local governments’ in this study [3]. Chiefs are important implementers of policies defined by these structures. District Assemblies are empowered to make by-laws “for the good rule and government of the whole or any part of the local government area”; for the implementation and monitoring of these by-laws, they cooperate with sub-district structures as well as chiefs. Procedures for the establishment, approval and enforcement of the by-laws are defined in the Local government Act [4].

Chiefs have been the intermediary institutions between rural citizens and government officials since colonialism and indirect rule. Traditional leadership consists of a hierarchy from village headman (VH) at village level to group village headman (GVH), traditional authority (TA), Senior TA and paramount chief. In this paper the terms ‘traditional authority’, and ‘chief’ are used for all these categories of chiefs. All these chiefs receive a honorarium by the state [1]. Customary law and authority are enshrined in the Constitution and the Chiefs Act empowers chiefs in rural settings to set by-laws, make judgments and to levy fines.

Health Centre Advisory Committees (HCAC) are established at every rural health centre; they act as a link between communities and health providers, deal with complaints about the health facility or health staff and co-sign for drugs delivered to the health centre. They constitute an important social accountability interface in the health sector [3].

**References**

1. O’Neil T, Cammack D. Fragmented governance and local service delivery in Malawi. ODI. 2014. https://www.odi.org/sites/odi.org.uk/files/odi-assets/publications-opinion-files/8943.pdf. Accessed 24 June 2016.
2. Commonwealth Local Government Forum. The local government system in Malawi. CLGF. www.clgf.org.uk/malawi. Accessed 24 June 2016.
3. NORAD. Local Perceptions, Participation and Accountability in Malawi’s Health Sector. Oslo. 2013. <https://www.norad.no/en/toolspublications/publications/2013/local-perceptions-participation-and-accountibility-in-malawis-health-sector/>. Accessed 3 February 2014.
4. Malawi Government. Local Government Act. Lilongwe: 1998. Local Government Act. Accessed 30 December 2017.
